# The Effect of Pretreatments on the Physical Properties and Starch Structure of Potato Chips Dried by Microwaves under Vacuum

**DOI:** 10.3390/foods11152259

**Published:** 2022-07-28

**Authors:** Aline Iamin Gomide, Ricardo Lemos Monteiro, Bruno Augusto Mattar Carciofi, João Borges Laurindo

**Affiliations:** Departament of Chemical and Food Engineering, Federal University of Santa Catarina, EQA/CTC/UFSC, Florianópolis 88040-900, SC, Brazil; aline.iamin.gomide@gmail.com (A.I.G.); ricardolemosmonteiro@hotmail.com (R.L.M.); bruno.carciofi@ufsc.br (B.A.M.C.)

**Keywords:** snacks, oil-free, healthy food, drying, starch

## Abstract

Native potato starch has a semi-crystalline structure associated with a low glycemic index. Microwave vacuum drying (MWVD) produces crispy snacks under mild temperatures, reducing starch structural changes. However, blanching pretreatment gelatinizes starch, reducing crystallinity. A promising alternative is drying raw or blanched-then-cooled potatoes by MWVD. Cooling the blanched potato before MWVD aims to promote the partial return of the crystalline structure. Thus, this study evaluated how different pretreatments affect potato chips’ starch structure and physical properties. Three samples were dried by MWVD: (i) raw (MWVD-RW), (ii) blanched (MWVD-BL), and (iii) blanched followed by cooling (4 °C for 48 h) (MWVD-BLC) potatoes. MWVD-RW samples presented a higher starch crystallinity (16.9%), which disappeared in MWVD-BL samples and partially returned in MWVD-BLC (8.7%). MWVD-BL and MWVD-BLC samples presented lower bulk (<0.338 g cm^−3^) density and higher porosity (>74%) and crispness. On the other hand, MWVD-BLC samples presented intermediate characteristics for color, true density, and porous distribution compared to others. All samples showed high porosity (>69%) and crispy texture. Therefore, based on the quality assessment, the MWVD-RW and MWVD-BLC produced healthy and crispy oil-free chips with a potentially lower glycemic index.

## 1. Introduction

Potato chips are a significant part of the snack food market in many countries. They are traditionally prepared by deep-frying raw or blanched potato slices in vegetable oils. This process results in physical and chemical alterations to the tissues and internal components of the cells, promoting a crispy crust formation and developing a delicious product [1,2]. Due to their unique sensory properties, potato chips have become the most popular snack for ordinary customers [3]. However, the excessive oil content concerns consumers, driving up searches for alternatives to produce oil-free potato chips or potato chips with reduced oil concentration [3,4].

Oil-free potato chips can be produced by dehydration. Microwave vacuum drying (MWVD) is a suitable process that operates at mild temperatures and results in crispy chips in shorter times than those observed for freeze- and air-drying [5,6,7,8,9,10,11].

Potato often undergoes blanching before dehydration (pretreatment). Blanching is a thermal treatment applied to raw vegetables before canning, freezing, or drying [12,13]. Hot water blanching is the most popular and commercially adopted method, as it is simple to establish and easy to operate [13]. Besides inactivating enzymes responsible for deterioration reactions of product quality, the blanching aims to modify the flavor and texture properties of the foods. For potato food, texture improvement is related to the formation of soluble pectic substances and starch structural changes during the heat treatment that decreases product firmness [13]. Starch’s changes are major determinants of its functional properties for food processing and formulation. In addition, they influence their physical properties, industrial applications, and digestibility, raising health concerns [14].

Starch is made of semi-crystalline granules composed of crystalline and amorphous regions arranged in a concentric growth ring [15]. This native structure is poorly digested in the human small intestine [16]. However, heating in the presence of excess water (as observed during blanching), causes starch gelatinization. The amorphous region absorbs water in this process, leading to starch granules swelling. This phenomenon destroys the starch crystallinity and its molecular organization. As a result, the granules become more accessible to digestive enzymes, which increases their glycemic index [17]. Consumption of rapidly digestible starch causes, in the long term, a series of health complications such as diabetes and cardiovascular diseases [18,19].

As digestibility is associated with starch structure, some techniques that modify its structure have been used to prepare slowly digestible starch [18]. Physical methods are widely used due to their safety, cost-effectiveness, and simplicity [18,20]. The most important process of the physical method is retrogradation following gelatinization. The gelatinized starch partially returns to its ordered native structure during retrogradation, which is less digestible [16,18]. Strategies such as cooling boiled potatoes have been applied to promote retrogradation and decrease the digestibility of potato starch [18,21,22,23].

Therefore, pretreatments that favor retrogradation can be associated with MWVD since this drying method promotes minimal starch changes when conducted below the gelatinization temperature, as reported by Gomide et al. [6], who conducted MWVD of raw potatoes. However, the pretreatments that favor retrogradation with MWVD have not been reported in the literature, and are deserving to be studied as an alternative to developing tasty and healthy food. Furthermore, the application of different pretreatments can modify the product’s physical properties, which is essential to understand because they can change the sensory acceptance.

In this context, the objective of this study was to evaluate the influence of different pretreatments on the starch structure and physical properties of potatoes dehydrated by microwave vacuum drying. Thus, three conditions were evaluated: (i) drying potatoes without pretreatment; (ii) drying blanched potatoes; (iii) drying blanched-then-cooled potatoes.

## 2. Materials and Methods

### 2.1. Samples and Pretreatments

Fresh potatoes (*Solanum tuberosum* L.) were selected by appearance, ensuring no external damage and microbial deterioration. First, selected potatoes were washed, manually peeled, and cut into 3.8 ± 0.5 mm thick slices by a mandolin (Progressive, Model-PL8^®^, Kent, WA, USA). Next, a stainless-steel cylindrical mold was used to cut the slices into a cylindrical shape (41.6 ± 0.1 mm in diameter) for uniformity. Then, three different procedures were applied before drying: (i) RW samples: raw potato slices were washed to remove the surface starch adhered after slicing and were placed on a filter paper (1 min) to remove the excess surface water; (ii) BL samples: the potato slices were blanched (95 ± 2 °C for 7 min) and cooled in an ice bath (6 ± 2 °C for 3 min) with a sample/water ratio of 1:20 (g:mL), and placed on a filter paper for 1 min; (iii) BLC samples: the slices were submitted to the same procedure as BL samples. After that, the samples were stored in a refrigerator (4 °C for 48 h) inside an impermeable package to avoid water loss.

### 2.2. Microwave Vacuum Drying Experiment

Potato samples were dried in a microwave vacuum dryer that used an inverter system to control the magnetron power outside and a rotatory vacuum chamber. Gomide et al. [6] describe this equipment in detail.

The oven (LG, Model-MS4297DIR A, Cajamar, SP, Brazil) has a volume of 42 L. The magnetron operates at 2.45 GHz with 1200 W as the maximum power. The microwave works by supplying the output power continuously (inverter), favoring control of the product temperature. The microwave was adapted to work uprighted, and the vacuum chamber (cylindrical polypropylene container) operated as a rotating drum to favor a uniform absorption of electromagnetic waves and heating. The drum was divided into four sections for product disposal to favor heating uniformity and prevent the product’s mechanical damage.

For the drying procedure, the samples (200 g) were uniformly positioned in the vacuum drum (50 g in each section), and the pressure of the chamber was set up to 4 kPa (measured by a transducer Warme, Model-WTP4010, Itaquecetuba, SP, Brazil) and maintained at this value during the entire drying process. Before turning on the microwave, the rotation speed was set to 2 to 3 rpm. Microwave energy input was gradually reduced during drying (1200-720-240-120 W), keeping the sample’s temperature below 60 °C. Power manipulation was used to obtain a high drying rate while preventing samples from not achieving the potato starch’s gelatinization range temperature, close to 69–76 °C [6], to avoid significant changes in the starch structure. Furthermore, it was guaranteed that in each moment of power reduction, the mass of the drying material was approximately equal in the three processes to ensure the same variation of power densities between them, enabling comparison.

The experimental data ensemble (samples weight, temperature, water activity) for each drying time was determined from a new drying experiment to avoid distortion of the results due to the interruption of the drying process. Immediately after turning off the oven, the potato temperature was determined by an infrared thermometer (Fluke, Model-62MAX, Everett, WA, USA). In this procedure, one measurement was taken from each drum section to calculate the average value. The power density (ratio between power and sample mass) was determined by weighting the samples (Knwaagen, Model-KNCD60/1, Cotia – SP, Brazil). Next, moisture content was determined by the gravimetric method using a vacuum oven at 70 °C (AOAC, 2005). Finally, the water activity was determined by a digital hygrometer (Decagon Devices Inc., Aqualab Model-Series 3, Pullman, WA, USA). The drying experiments were performed in two repetitions for each experimental point.

The curves of the temporal evolution of moisture content and sample mass were represented by fitting the Midilli, Kucuk, and Yapar [24] model to the experimental data. The values of the fitted mass were used to determine the evolution of power density. In addition, a linear equation was fitted to the first part of the experimental drying curve to verify the existence of a constant drying rate period.

The drying experiments were conducted using the same parameters and procedures reported by Gomide et al. [6], who evaluated the impact of power density on the physical characteristics and acceptability of chips produced from raw potato slices. Thus, their experimental data were compared with those obtained in the present study for potato chips produced from pretreated samples.

### 2.3. Starch Granules Structure

The starch structure was evaluated for samples before (RW, BL, and BLC) and after the MWVD process (MWVD-RW, MWVD-BL, and MWVD-BLC) by light polarized microscopy, scanning electron microscopy, and X-ray diffraction. Samples were freeze-dried and ground to a powder before analysis.

#### 2.3.1. Light Polarized Microscopy (PLM)

Light polarized micrographs were obtained with a confocal microscope (Leica, Model-DMI6000 B, Wetzlar, Hesse, Germany) equipped with a polarizing filter. The micrographs show whether starch granules presented birefringence, identified from the Maltase cross, indicating a semi-crystalline structure and molecular organization.

#### 2.3.2. Scanning Electron Microscopy

A scanning electron microscope (JEOL, Model-JSM 6390LV, Tokyo, Japan) operating under 10 kV was used to investigate the starch granule shape and changes on the sample surfaces. Samples were coated with a fine gold layer in an anion-sputtering apparatus (EM SCD500, LEICA). Micrographs were captured at 500 and 1500 times magnification.

#### 2.3.3. X-ray Diffraction

The X-ray diffraction patterns and the relative crystallinity of samples were determined by an X-ray diffractometer (Rigaku, Model-MiniFlex600, Osaka, Japan), working at 40 kV and 15 mA with Cu-Kα1 and Cu-Kα2 radiation source. The analysis was performed in the scattering range (2θ) of 4–40°, with a scanning rate and step size of 2°min^−1^ and 0.05, respectively. The relative crystallinity (RC) was calculated as described by Singh, Dartois, and Kaur [25] using Equation (1):

(1) $$\mathrm{RC}\;=\frac{\mathrm{Ac}}{\mathrm{Ac}\;+\;\mathrm{Aa}}\times 100,$$

in which Ac and Aa are the areas of the crystalline and amorphous phases, respectively.

### 2.4. Physical Properties of Dried Samples

#### 2.4.1. Optical Micrographs

Optical micrographs were captured from manually fractured samples using an optical microscope (Meiji, Model RZ, Miyoshi, Japan) coupled to a microscopic câmera (Opticam OPT 10000, Chácara Santo Antônio, SP, Brazil). The captured images were analyzed using TSview (Tucsen, V, 7.3.1.7, Fuzhou, China).

#### 2.4.2. Color Measurements

A computer vision system was used to determine the color parameters of samples, according to that described by Cárdenas-Pérez et al. [26] with minor adaptations. Images taken from a digital câmera (Nikon Corporation, Model D5500, Tokyo, Japan) were treated using the ImageJ 1.6.0 software (National Institutes of Health, Bethesda, MD, USA). The colors were converted from the RGB system to CIELab scale using the color-space converter plug-in.

#### 2.4.3. Bulk Density, True Density, and Porosity

The bulk density was obtained from the ratio between the mass of sample and bulk volume, which was determined by measuring the buoyant forces of potatoes immersed in n-heptane [27,28]. A helium gas pycnometer (Micrometrics, Model-AccupPyc II 1340, Norcross, GA, USA) was used to determine the true density. Porosity was obtained from bulk and true density, according to Carciofi, Prat, and Laurindo [29].

#### 2.4.4. Acoustic–Mechanical Properties

Puncture tests were performed in a texture analyzer (Stable Micro System, Model-TA-HD-Plus, Godalming, Surrey, UK). A cylindrical probe (2 mm diameter) penetrated to 90% of the original sample thickness at 1 mm s^−1^. The following mechanical parameters were determined: (a) the area under the curve (force versus time)—work performed; (b) the force peaks number (force drops higher than 0.049 N); (c) maximum force; (d) average peak force.

An acoustic sensor (GRAS Sound & Vibration, Model-GRAS 46AE ½” CCP Free-field Microphone, Holte, Denmark) recorded the sound emitted during penetration tests. The texture analyzer and the microphone (positioned 5 cm apart at a 45° angle to the sample) were placed inside a semi-anechoic chamber to reduce the background noise, as described by Andreani et al. [30]. The results were treated with a band-pass FIR filter, with a frequency between 1 kHz and 22 kHz, as Moraes et al. [31] described. The acoustic parameters were: (a) acoustic peaks number (drops of sound pressure level higher than 10 dB); (b) the sound pressure level avarege considering the ten higher peaks (SPL_10_); (c) the maximum sound pressure level (SPL_max_). The data of mechanical (force versus time) and acoustic (sound pressure level versus time) analysis were synchronized using Matlab^®^ 7.13 (Math-Works Inc., Model-R2011b, Natick, MA, USA).

### 2.5. Statistical Analysis

The results of color, bulk, and true densities, porosity, and acoustic–mechanical properties were statistically analyzed using Statistica 7.0 (StatSoft, Tulsa, OK, USA), using the analysis of variance (ANOVA), followed by the Tukey test (0.05 significance level). 

## 3. Results and Discussion

### 3.1. Drying Kinetics

Raw potato samples (RW) presented a moisture content ($X_{\mathrm{db}}$) of 5.746 g g^−1^ ± 0.310 g g^−1^ (dry basis-db). After pretreatment, $X_{\mathrm{db}}$ increased to 6.303 g g^−1^ ± 0.513 g g^−1^ (blanched, BL) and 6.303 g g^−1^ ± 0.662 g g^−1^ (blanched-then-cooled, BLC), due to water incorporation during immersion blanching. Monteiro et al. [9] also reported an increase in the moisture content of sweet potato slices after blanching. As BLC samples were stored under refrigeration in an impermeable package, the water loss to the cooling air was avoided, keeping its $X_{\mathrm{db}}$ approximately the same reported for BL samples.

**Figure 1:**
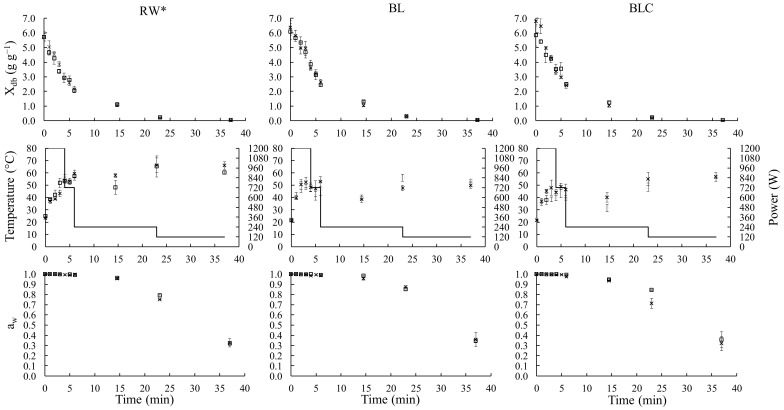
Variation of moisture content (*X*_db_), temperature, microwave power (▬) and water activity (a_w_), during the two drying repetitions of RW, BL, and BLC samples. (□,×) Symbols indicate the duplicate of the drying process. * Data from Gomide et al. [6].

**Figure 2:**
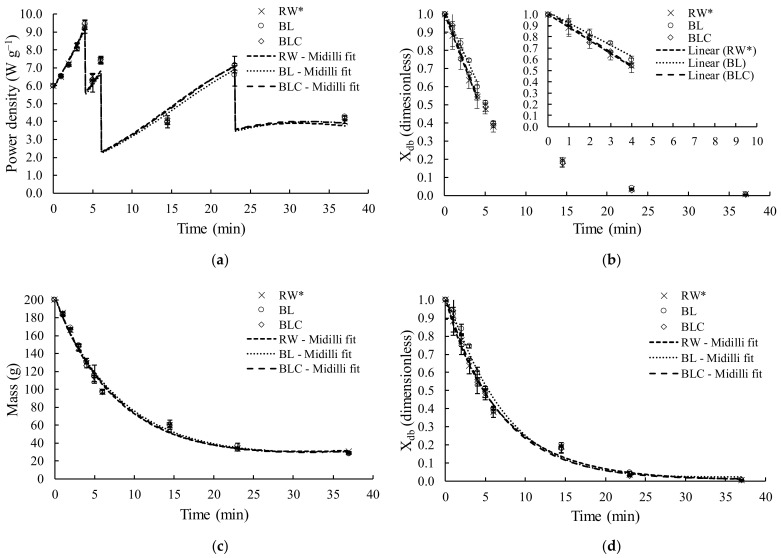
Temporal evolution of power density (**a**), constant drying rate period, represented by the straight line fit (**b**), the temporal evolution of mass (**c**), and temporal evolution of dimensionless moisture (**d**). Midilli model showed the goodness of fit (R^2^ > 0.9). * Data from Gomide et al. [6].

The temporal evolution of $X_{\mathrm{db}}$, water activity, temperature, and microwave power during the drying of RW, BL, and BLC samples are presented in Figure 1. The data are in duplicate for each experimental point and had excellent reproducibility. Over time, the microwave power was reduced in steps to keep the drying temperature under 60 °C. At each step of reduction, all drying processeses had the same mass (Table 1) and, consequently, the same pattern of power density variation (Figure 2a).

During the first four minutes of drying (period in which the maximum microwave power was used, 1200 W), the moisture of all the samples presented linear behavior (Figure 2b), which was corroborated by the fit of the straight line to experimental data (R^2^ > 0.99), as reported by Monteiro et al. [8,32,33] during microwave vacuum drying of bananas, tomatoes, chickpea, and carrots. The beginning of the drying was marked by a high water content and constant water activity (≈1). At that moment, constant power input was constantly applied (1200 W), and parts were dissipated and converted into latent heat for vaporization of free water, keeping the liquid–vapor transition constant [34,35,36]. The period of constant drying rate corresponded to only ten percent of the total drying time and was responsible for removing fifty percent of the samples’ initial moisture. After that, the mass transfer resistance increased, the water vapor pressure decreased, and the microwave power gradually reduced, leading to the slow moisture remotion [5,8,35].

The pretreatments did not affect the drying kinetics, as observed in Figure 2c,d, which show the time evolution of mass and dimensionless moisture (db) of potato samples. Additionally, after the same drying length (37 min), the dried samples presented very similar moisture (~0.045 g g^−1^) and water activity (~0.33). Furthermore, the drying rate at the constant period was significantly equal (*p* > 0.05) for the three different pretreatments of samples, which were 0.67 g s^−1^ ± 0.04 g s^−1^ (RW), 0.60 g s^−1^ ± 0.09 g s^−1^ (BL), and 0.74 g s^−1^ ± 0.08 g s^−1^ (BLC).

### 3.2. Starch Granule Structure

#### 3.2.1. X-ray Diffraction

**Figure 3:**
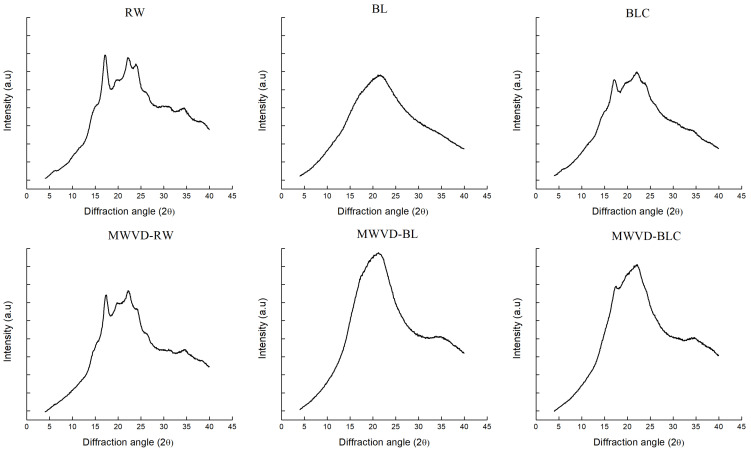
X-ray diffraction pattern of fresh (RW, BL, BLC) and dried (MWVD-RW, MWVD-BL, MWVD-BLC) potato samples.

X-ray diffraction patterns of the different samples before (RW, BL, and BLC) and after (MWVD-RW, MWVD-BL, and MWVD-BLC) drying are shown in Figure 3. This technique was applied to identify the samples’ crystallinity and determine their relative crystallinity. Furthermore, it was possible to determine the diffraction pattern to obtain crystal arrangement.

X-ray detects long-range ordered structures involving a regular and repeated arrangement of double helices [14], identified by peaks, as observed in RW samples. The raw sample presented a relative crystallinity (RC) of 22% and showed a B X-ray diffraction pattern, typical of regular potato starch [37], with reflection intensities at 2θ values of 15°, 19°, 22° e 24°, reflecting a three-dimensional order and crystallinity of native potato starch granules. Similar results were reported by Colussi et al. [38] and Tian et al. [39] for native potato starch. The peaks disappeared for blanched samples (BL), suggesting starch gelatinization. During heat processing, the water molecule mobility to the amorphous regions is facilitated. These molecules expand and transmit disruptive forces to the crystalline regions, destroying their structure [39,40]. However, some peaks appeared in blanched-then-cooled samples, which presented reflection intensities at 2θ values of 17° and 22° and RC of 13.8%. Tian et al. [39] reported similar values after cooling boiled potatoes at 4 °C for 24 h. These results suggest that retrogradation occurred during cooling storage, promoting a partial return of crystalline order.

The diffraction pattern of the dried sample presented similar behavior, where peaks were observed only for MWVD-RW and MWVD-BLC samples. However, the dried samples showed fewer peaks and lower RC values than the fresh samples (RW and BLC). Despite the variations reported, the RC values observed for dried samples (16.9% for MWVD-RW and 8.7% for MWVD-BLC) presented a low decrease compared to the samples before drying. The result shows the potential of MWVD to preserve starch crystallinity.

#### 3.2.2. Light Polarized Microscopy and MEV

**Figure 4:**
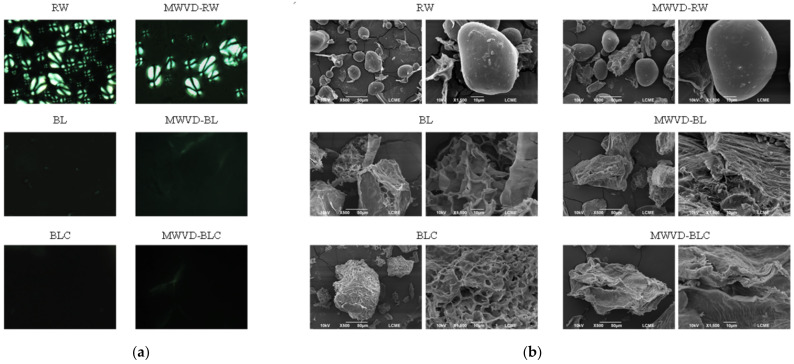
Micrographs obtained by polarized light microscopy (**a**) and MEV-500× and 1500× magnification (**b**) of fresh (RW, BL, and BLC) and dried (MWVD-RW, MWVD-BL, and MWVD-BLC) potato samples.

Micrographs obtained by light polarized microscopy and MEV are presented in Figure 4. RW samples presented birefringence (Figure 4a), marked by various structures similar to the maltese cross, indicating typical crystallinity of native starch granule [41]. The frequency of these structures was reduced in MWVD-RW samples, attributed to gelatinization during drying. However, the birefringence phenomenon was still pronounced, indicating that the MWVD process did not strongly affect the starch granule structure. The SEM images of RW and MWVD-RW samples (Figure 4b) corroborate these results, showing round and elliptical granules with a smooth surface (without fissures), typical of native potato starch [18].

Birefringence disappeared in BL, BLC, MWVD-BL, and MWVD-BLC samples (Figure 4a) due to gelatinization triggered by blanching. Although cooling provided partial crystallinity to BLC and MWVD-BLC (as seen in DRX analysis results), these samples did not show birefringence since the loss of this property is an irreversible change. Retrogradation caused starch molecules to re-associate into an ordered structure, different from native starch [42]. Consequently, starch granules were no longer observed in pretreated samples (BL, BLC, MWVD-BL, and MWVD-BLC) (Figure 4b). During blanching, gelatinization promotes a significant change in starch structure. The starch granules swell, melt and fuse to form a netlike structure, totally collapsing the cell. During this process, the granules lose their identity, and the beams of the neighboring deformed granules join together [18,39]. Finally, a sponge-like structure with cavities was formed in the BL sample. BLC samples showed a more compact structure with smaller cavities, resulting from an extensive aggregation of the granule fragments (amylose and amylopectin) promoted by retrogradation. Similar results were reported by Colussi et al. [38] and Xie et al. [18] after cooling boiled potatoes.

The MWVD resulted in a significant change in sample structure observed by SEM micrographs. MWVD promotes rapid evaporation resulting in pores surrounded by a more compact structure. As SEM was conducted in powder samples, most of the portion captured by micrographs corresponds to a region of a denser solid matrix, hindering the characteristics resulting from the pretreatment.

### 3.3. Physical Properties of Dried Samples

#### 3.3.1. Optical Micrographs

**Figure 5:**
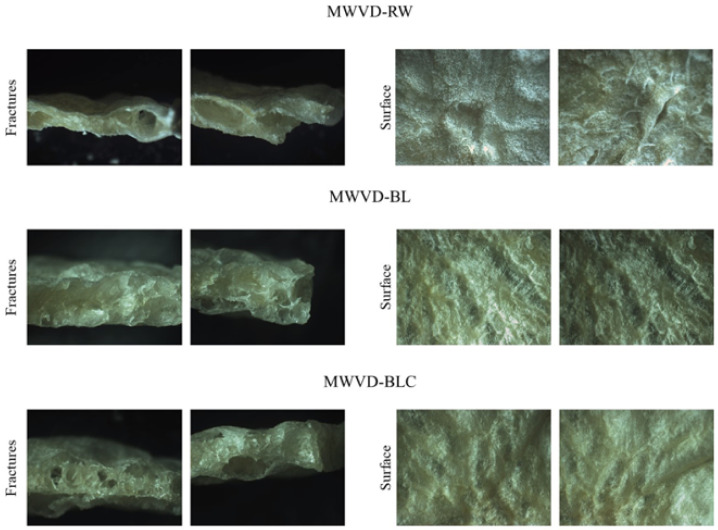
Optical micrographs of fractures and surface of dried samples MWVD-RW, MWVD-BL, and MWVD-BLC.

The optical micrographs of fractures and the surface of dried samples are shown in Figure 5. All samples presented an expanded and porous structure due to the puffing effect caused by the vacuum pressure and the volumetric heating generated by microwaves [8,9,35]. However, a more expanded structure marked pretreated samples (MWVD-BL and MWVD-BLC). The blanching treatment favors expansion, which reduces firmness and increases the softness of the cell structures of vegetables due to gelatinization and the formation of soluble pectic substances [13,43].

Pretreatment also affects the porous structure. During gelatinization, hydrogen bonds between amylose and amylopectin (intrachain and interchain) are broken, and water molecules bond to the exposed hydroxyl groups [15]. Consequently, a network (starch–water) was formed where water molecules became evenly distributed and surrounded by starch molecules. Thus, water evaporation may have taken place at various points in the structure during drying, resulting in a smaller porous structure uniformly distributed in the sample MWVD BL. On the other hand, a starch–water network was not formed in MWVD RW samples since gelatinization occurred to a lesser extent. Therefore, the water may have concentrated in certain micro-regions, where evaporation occurred preferentially, originating in large and dispersed porous samples. MWVD BLC samples presented intermediate characteristics: a uniformly distributed porous structure due to gelatinization and larger and isolated pores due to retrogradation. Retrogradation is marked by starch molecules’ reassociation and water release [16]. Therefore, the water released may have concentrated in specific regions, creating larger pores during MWVD.

The surface images show the surface irregularities, which reflect the pore characteristics of samples, marked by larger pores in MWVD-RW, smaller pores in MWVD-BL, and their combination in MWVD-BLC.

#### 3.3.2. Color Measurements

**Figure 6:**
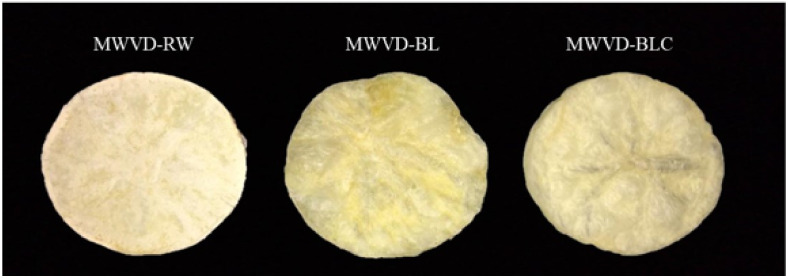
Digital image of dried samples MWVD-RW, MWVD-BL, and MWVD-BLC.

Color differences between samples can be observed in Figure 6 and confirmed by the color parameters (Table 2). Pretreatment significantly changed (*p* < 0,05) $a^{*}$ and $b^{*}$ parameters, while no differences (*p* > 0.05) were detected in $L^{*}$. The non-pretreated sample (MWVD-RW) presented non-gelatinized starch on its surface, resulting in a floured aspect (Figure 6), which could have contributed to some color differences. Furthermore, heat treatment results in a cellular collapse and degradation of heat-sensitive compounds, such as anthocyanins, a natural pigment present in potatoes [23,44,45]. This event can explain the MWVD-BL and MWVD BLC color changes. Some studies also reported changes in the color parameters of potatoes as the heat treatment became more intense [44,46,47]. Furthermore, anthocyanins may have leached into water during blanching since they are highly water-soluble pigments [23]. In addition, the gelatinized starch with a disordered structure could have altered the light reflection on the MWVD BL surface, contributing to further color differences. Otherwise, MWVD-BLC samples showed color parameters closer to MWVD-RW, probably due to the partially reordered starch resulting from retrogradation.

#### 3.3.3. Bulk Density, True Density, and Porosity

Table 2 shows data on the bulk density ($\rho_{b}$), true density ($\rho_{t}$), and porosity of dried potato samples. All samples presented high porosity (close to 70%), similar to the results reported by Monteiro et al. [9] and Barreto et al. [5] for potato and sweet potato dehydrated by MWVD, respectively. During the MWVD, the increase of the capillary pressure, which causes shrinkage, is compensated by forces related to gas expansion caused by volumetric heating under vacuum, producing highly porous dehydrated products [9,48]. The MWVD significantly increased sample porosity (*p* < 0.05) of pretreated potatoes (MWVD-BL and MWVD-BLC). The blanching, accompanied by tissue softening, favored the expansion of pretreated samples during drying (as shown in optical micrographs), favoring the porous space formation.

The MWVD-BL and MWVD-BLC samples showed significantly (*p* < 0.05) lower values of $\rho_{b}$ as a consequence of their higher air volume proportion (porosity). The true density $\rho_{t}$ depends only on the water content and solid type, excluding air pores [49]. Thus, the $\rho_{t}$ differences between samples are explained by the solid matrix structure. The destruction of the crystallinity during gelatinization is accompanied by the formation of the starch–water network and the loss of the starch’s highly ordered packaged structure. This event led to an increase in the true volume of MWVD-BL, justifying the significantly (*p* < 0.05) lower values of $\rho_{t}$. On the other hand, no significant difference was detected between MWVD-BLC and the other samples (*p* > 0.05), probably due to the partial reassociation of starch molecules during retrogradation, resulting in partial molecular reordering.

#### 3.3.4. Acoustic–Mechanical Properties

**Figure 7:**
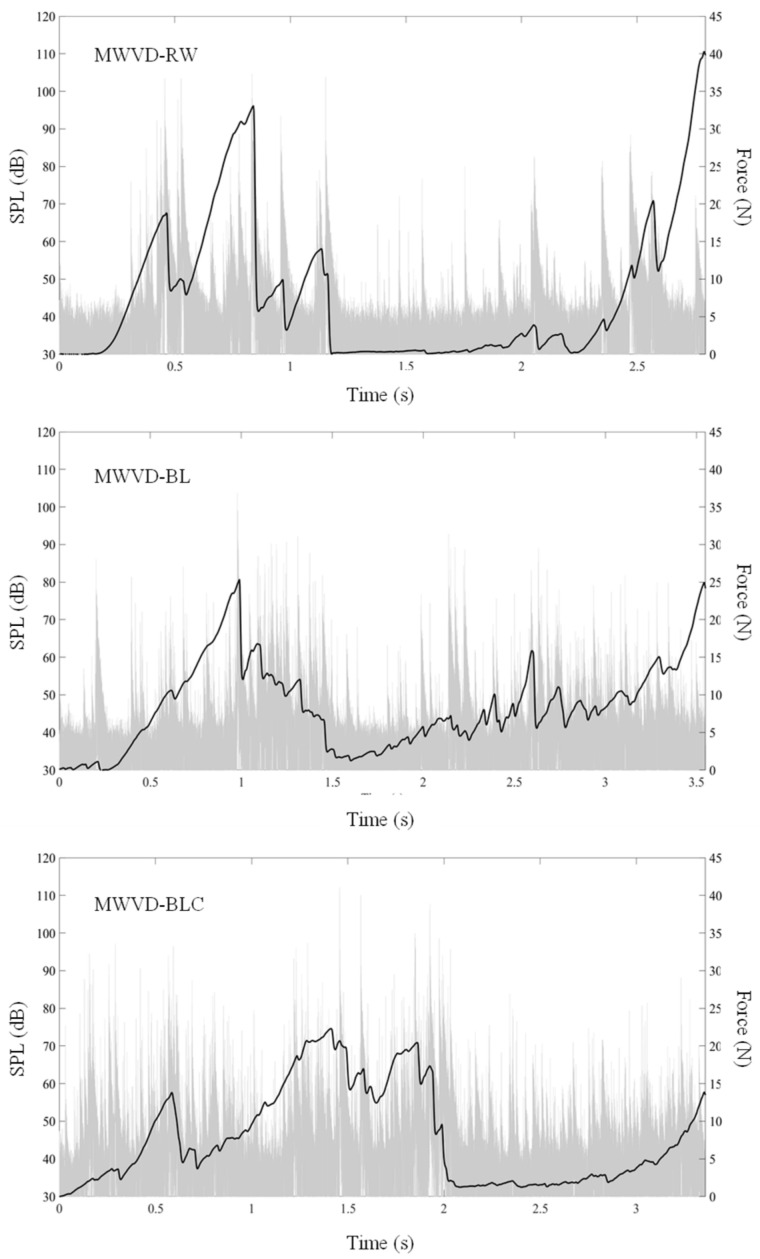
Representative curves of mechanical (▬) and acoustic (sound pressure level-SPL) (▬) data of dried samples (MWVD-RW, MWVD-BL, and MWVD-BLC).

Figure 7 shows the mechanical (force versus time) and acoustic (sound pressure level versus time) curves, and Table 3 presents the determined parameters. All samples resulted in jagged force-deformation curves (Figure 7) with a high number of acoustic and force peaks (Table 3), typical behavior of crispy products [1,50,51]. Curve jaggedness depicts different fracture events and reflects the progressive collapse of the porous and brittle structure of low deformation during the probe penetration. The sample crackling is accompanied by sound emission [1,5,9,52,53]. This behavior was observed in crispy snacks, such as bananas [31], sweet potatoes [11], restructured pineapple [54], and mango leathers [55].

The jagged pattern was more pronounced in MWVD-BL and MWVD-BLC curves, as confirmed by its higher number of acoustic and mechanical peaks, suggesting a crispier texture when compared to the MWVD-RW sample. This result can be explained by the higher porosity associated with the presence of smaller pores (observed by optical micrographs). Thus, as the probe penetrates the sample, it goes through more pores in its path, resulting in more force events with sound emission.

The number of force peaks observed for MWVD-BL and MWVD-BLC samples was 1.34-times greater than that observed for MWVD-RW samples. Concerning the acoustic peaks, this proportion increased to 2.45. The highest acquisition frequency of acoustic data and the microphone sensitivity make this methodology very sensitive to detecting structural differences among the different samples. Figure 7 illustrates the differences between the mechanical and acoustical signatures of the three samples submitted to the puncture test. Some force events may have occurred but not been detected, showing the complementary importance of acoustic tests in describing the instrumental texture. Andreani et al. [30] also reported this occurrence in penetration tests of cereal bars, and as a result, sensory properties were more correlated with acoustic rather than mechanical properties.

Generally, samples showed low mean force, maximum force, area values, and high SPL10 and SPLmax, typical of brittle structures of crispy texture products [10,54]. The increases in both area and the maximum force are associated with the hardness of the material felt by panelists [56], which reinforces the formation of porous and fragile sample structures. A higher number of force peaks was directly related to the higher number of acoustic peaks, SPL10 and SPLmax [1,10,57,58], corroborating the present findings. Pretreatments did not affect mean force, maximum force, area, SPL10, and SPLmax parameters (*p* > 0.05). The MWVD-RW sample showed high standard deviations, close to the mean value of these parameters, indicating significant differences in detection. The high values are explained by the heterogeneity of the samples’ microstructures, which had dispersed large pores (shown by optical micrographs). Fewer force events are detected when the probe goes through a large pore. Otherwise, more force events are detected when perforation goes through many small pores. Thus, the results are dependent on the perforation path, increasing the standard deviation.

## 4. Conclusions

Raw potato has a significant crystallinity related to its native starch granules, which are completely destroyed by heating and starch gelatinization during blanching. However, this crystallinity partially returns in blanched-then-cooled samples during refrigerated storage due to starch retrogradation.

A significant result is that the microwave vacuum drying process, as performed in this study, caused few changes in the starch crystallinity of the raw and pretreated samples. Therefore, it is possible to produce potato chips with significant crystallinity from the microwave vacuum drying of raw and blanched-then-cooled potato slices. This is crucial to produce potato chips with a reduced glycemic index.

The pretreatments also affected the physical structure of potato chips. Chips produced from pretreated samples had higher porosity and crispness and lower bulk density than those produced from raw samples. In addition, the retrogradation provided intermediate characteristics to the blanched-then-cooled potatoes for color, true density, and porous distribution. Although there were differences, all the dried samples presented a highly porous structure with a crispy texture, one of the most appealing quality attributes for consumers of potato chips.

Thus, microwave vacuum drying of raw or blanched-then-cooled potatoes potentially addresses sensory and health issues simultaneously, delivering high-quality snacks without added oil and with higher starch crystallinity, which likely results in a lower glycemic index.

## Figures and Tables

**Table 1:** Mass (g) of samples and power density proportion between the drying process of each sample (RW, BL, and BLC) during each power reduction step.

Power Reduction Step	Mass (g)			Power Density Proportion	
	RW	BL	BLC	RW:BL	RW:BLC
Drying start	200 ± 0 ^a^	200 ± 0 ^a^	200 ± 0 ^a^	1.0	1.0
1200 W—720 W	129 ± 5 ^a^	131 ± 1 ^a^	127 ± 2 ^a^	1.0	1.0
720 W—240 W	97 ± 3 ^a^	98 ± 2 ^a^	97 ± 2 ^a^	1.0	1.0
240 W—120 W	34 ± 1 ^a^	36 ± 4 ^a^	34 ± 2 ^a^	1.1	1.0

^a^ Means with the same letter in the lines indicate no significant differences (*p* > 0.05) by the Tukey test.

**Table 2:** CIELab color parameters ($L^{*},\;a^{*},b^{*}$), true density ($\rho_{t}$), bulk density ($\rho_{b})$ and porosity (ε).

	Samples		
	MWVD-RW **	MWVD-BL	MWVD-BLC
$L^{*}$	86.37 ± 1.06 ^a^	85.04 ± 1.44 ^a^	84.67 ± 1.91 ^a^
$a^{*}$	−2.87 ± 0.15 ^a^	−3.41 ± 0.20 ^b^	−2.81 ± 0.20 ^a^
$b^{*}$	11.94 ± 0.75 ^c^	27.13 ± 1.41 ^a^	15.66 ± 0.62 ^b^
$\rho_{t}$ (g cm^−3^)	1.499 ± 0.071 ^a^	1.267 ± 0.035 ^b^	1.325 ± 0.004 ^ab^
$\rho_{b}$ (g cm^−3^)	0.466 ± 0.077 ^a^	0.308 ± 0.031 ^b^	0.338 ± 0.013 ^b^
ε (%)	69.0 ± 3.7 ^b^	75.7 ± 1.8 ^a^	74.5 ± 0.9 ^a^

^a,b,c^ Means with different letters in the same line indicate significant differences (*p* < 0.05) by the Tukey test. ** Adapted from Gomide et al. [6].

**Table 3:** Parameters obtained in the acoustic–mechanical test for dried samples (MWVD-RW, MWVD-BL, and MWVD-BLC).

Parameters		Samples		
		MWVD-RW *	MWVD-BL	MWVD-BLC
Mechanical	Area (*n*.mm)	13.4 ± 12.2 ^a^	17.1 ± 8.6 ^a^	11.9 ± 6.2 ^a^
	Number of force peaks	23 ± 8 ^b^	31 ± 8 ^a^	31 ± 11 ^a^
	Average Peak force (*n*)	7.1 ± 5.0 ^a^	6.9 ± 2.1 ^a^	5.2 ± 1.8 ^a^
	Maximum force (*n*)	25.5 ± 22.2 ^a^	21.8 ± 9.3 ^a^	16.8 ± 5.2 ^a^
Acoustic	SPL_10_ (dB)	103 ± 5 ^a^	103 ± 6 ^a^	105 ± 4 ^a^
	SPL_max_ (dB)	108 ± 6 ^a^	108 ± 7 ^a^	111 ± 5 ^a^
	Number of acoustic peaks	2374 ± 868 ^b^	5557 ± 1591 ^a^	6091 ± 2155 ^a^

^a,b^ Means with different letters in the same line indicate significant differences (*p* < 0.05) by Tukey test. *Adapted from Gomide et al. [6].

## Data Availability

Data are contained within the article.
